# Food neophobia among university students in Saudi Arabia: a cross-sectional survey of prevalence and predictor analysis

**DOI:** 10.3389/fpubh.2025.1571899

**Published:** 2025-04-01

**Authors:** Najim Z. Alshahrani

**Affiliations:** Department of Family and Community Medicine, Faculty of Medicine, University of Jeddah, Jeddah, Saudi Arabia

**Keywords:** food neophobia, eating habits, eating disorder, food preference, students

## Abstract

**Introduction:**

Due to the absence of scientific information on food neophobia (i.e., fear or reluctance to try new or unfamiliar foods) among young adults in Saudi Arabia, the aim of this study was to assess the prevalence and predictors of food neophobia in Saudi university students.

**Methods:**

This cross-sectional study included 480 students from two public universities of Saudi Arabia. The data was collected via an internet-based structured questionnaire. Participants’ sociodemographic, health and behavioral information were included as explanatory variables (15 variables). Participants’ food neophobia was assessed using a10-item validated food neophobia scale. A logistic regression model was fitted to find out the predictors of food neophobia.

**Results:**

Approximately half of the participants (49.6%) exhibited food neophobia. Participants who engaged in regular physical exercise had a lower risk of developing food neophobia than their counterparts (AOR: 0.43, 95%CI: 0.20–0.92). Participants with food allergies (AOR: 4.36, 95%CI: 2.73–6.94) and disordered eating attitudes (AOR: 2.52, 95%CI: 1.27–5.02) and who took dietary supplements (AOR: 6.76, 95%CI: 3.54–12.90) were more likely to be food neophobic. Moreover, participants’ preferences for fish and sea food (r_s_ = −0.150), milk and dairy products (r_s_ = −0.309,), chocolate and candies (r_s_ = −0.329) and snacks, chips, and nuts (r_s_ = −0.166) were significantly correlated with food neophobia.

**Conclusion:**

A higher level of food neophobia was observed among surveyed university students in Saudi Arabia. Several factors, such as regular physical exercise, food allergies, dietary supplement consumption, and disordered eating attitudes, were found to be associated with food neophobia. To provide empirical evidence on food neophobia, additional research with large and representative samples could be performed in other regions of Saudi Arabia.

## 1 Introduction

Certain types of eating disorders, such as anorexia nervosa, bulimia nervosa, and binge eating, are associated with body dissatisfaction, body weight, body perception, and overeating; while others (such as restrictive food intake disorder) are marked by disinterest in food and avoidance of its sensory attributes, such as odor and visual appeal (1–3). These eating disorders affects food choices and pose adverse impact on physical health, social functioning, and quality of life (3, 4). One form of behavioral and personality trait that affect food choices and preferences is often known as food neophobia (5, 6).

Neophobia refers to the fear or dislike of new things, experiences, or changes. It is a psychological phenomenon where people feel hesitant or anxious when faced with unfamiliar situations, objects, or concepts. The word originates from the Greek terms “neo” (meaning new) and “phobia” (meaning fear). Pliner and Hobden (7) described food neophobia as an unwillingness to eat and/or resist new foods. It is an actual observable behavior and an inevitable component of character traits with significant hereditability (8, 9). Food neiophobia is a combination of biological and behavioral mechanism that an individual adapted or developed to protect themselves from consuming harmful foods (10). As food safety is ensured, and behavioral nutrition and nutritional epidemiology have developed rapidly in recent decades, individuals should consider the nutritional and health benefits of consuming a diverse range of foods. A food neophobic condition can deprive the individual of access to nutritious foods due to the avoidant nature of new foods. Evidence shows that this condition leads to low dietary diversity and poor diet quality, and are associated with the risk-factors of negative health consequences including diabetes, obesity etc. (11–14).

Food neophobia has been thoroughly researched in children across the world (15, 16), but little is known about its prevalence and contributing factors in young adult population. The number of studies targeting food neophobia among university students has increased recently because of their increased susceptibility to eating disorders and unhealthy eating habits. According to a recent global review study covering 40 countries, disordered eating affects 20% of university students (17). University students in China (18) and Bangladesh (5) have been shown to exhibit higher rates of food neophobia, as indicated by recent studies. Several factors such as gender, family income, body mass index (BMI), food allergy, long-term nutrition course and family eating patterns were appeared to affect the university students’ neophobia to food (5, 17–19).

In Saudi Arabia (a Middle East country), the population has morbidity due to lifestyle, metabolic risk factors and dietary habits (despite of improvement in healthcare access and quality) (20). It is concerning because non-communicable diseases (NCDs) account for nearly three-quarters (73%) of all mortality in Saudi Arabia as well as rising healthcare burden (21). A healthy and diversified diets play significant role in preventing NCDs and malnutrition; therefore, behavioral changes in food intake can be an effective approach to get nutrition-rich foods. However, the snacking patterns and eating habits of Saudi university students are mostly unhealthy, leading to potential risk factors for overweight and obesity (22–25). Furthermore, a latest study estimated that 40% of participating university students had poor nutrition literacy in Saudi Arabia (26). Considering university students a vulnerable group in terms of dietary intake, nutrition literacy and eating disorder (27), an investigation on food neophobia is needed to explore the topic in the Saudi context.

### 1.1 Objective(s) and research question

The objective of this study was to assess the prevalence and predictors of food neophobia among university students in Saudi Arabia. This study was guided by three key questions:

- What is the prevalence of food neophobia among Saudi university students?
- What sociodemographic, health and behavioral factors are associated with food neophobia?
- What connection exists between food neophobia and food preference?

## 2 Materials and methods

### 2.1 Data source

The typical primary data sources for this project were two Saudi Arabian public higher education institutions: University of Jeddah and King Faisal University. The University of Jeddah is located in western region of the country, specifically at Jeddah city under the province of Makkha. The King Faisal University is situated at Hofuf city (Saudi Arabia’s Eastern province). A large number of students from different regions of Saudi Arabia are studying in these well-renowned institutions. Both institutions offer bachelor and post-graduate degrees in multidisciplinary fields such as science, engineering, education, etc. These institutions are the country’s top-ranked and rapidly expanding research and educational establishments, which supports the rationale of conducting such study among their students.

### 2.2 Study type and ethical compliance

This study was cross-sectional in nature and conducted over a six-month period, particularly from May 2023 to February 2024. The ethical clearance was taken from the Research Ethics Committee of King Faisal University, Saudi Arabia (reference number: KFU-REC-2022-FEB-EA000431). Informed consent was taken from all the surveyed individuals after clarifying the study objectives. Participants were guaranteed that the information would only be utilized for research purposes and that involvement would not affect their academic standing in any way.

### 2.3 Participants and eligibility criteria

Both undergraduate and post-graduate levels students of the selected universities (n = 2) were included as study participants. The criteria for inclusion of students as study participants were as follows: (a) they had to be adults and Saudi nationals, and (b) they had to be current student at the time of data collection. This study did not include students who suffered from chronic complications and clinically-diagnosed psychological problems including depression. The exclusion criteria were established to reduce the risk of underestimating or overestimating the outcomes, as these conditions may affect dietary intake.

### 2.4 Survey procedures and sampling

An internet-based structured questionnaire was used to gather data (Supplementary File 1). The online survey was constructed with Google Docs and the survey link was circulated to the chosen institutions’ students. Students were invited to the survey using their e-mail address (institutional). The e-mail body indicated the reason for the invitation and attached a survey summary document outlining the study objectives, inclusion and exclusion criteria, and ethics and consent-related information. Study participants were recruited using a list-based sampling frame strategy. Students’ e-mail lists were obtained from the register/admission office of the respective institution, and 750 samples were picked for survey invitations using a computer-generated random selection (28). It should be noted that survey invitations were sent to twice the required sample size.

This study followed Cochran’s formula to get a statistically adequate sample size (29). A minimum sample of 384 students was computed by accounting for a 50% prevalence of food neophobia (*p* = 0.5), a 95% reliability level (Z = 1.96), and a 5% allowable sampling error (e = 0.05). Since there was no evidence available regarding food neophobia among Saudi university students. This study used the 50% population proportion as a guide. After filtering out missing data, 480 samples were finally included in the statistical analysis.

### 2.5 Description of the study variables

Participants’ sociodemographic, health and behavioral information were included as explanatory variables (15 variables). Participants’ sociodemographic information including gender (male or female), age (18–21 or 22–25 or > 25 years), study discipline (medicine or education or engineering or science), parent education level (illiterate or elementary or intermediate or secondary or university), current living area (rental house or dormitory or own house) and monthly family income (<5,000 or 5,001–10,000 or 10,001–15,000 or > 15,000 SAR) were obtained (07 variables). A total of 08 variables related to health and behavior like self-reported BMI, regular physical exercise (yes or no), smoking status (yes or no), food allergy (yes or no), nutritional anemia (yes or no), experience of illness after having new foods (yes or no), dietary supplements (yes or no) and disordered eating attitudes (yes or no) were included.

The Eating Attitude Test-26 (EAT-26) questionnaire, which has been widely validated, was used to measure disordered eating attitudes (30). This study employed a validated Arabic version of EAT-26, and earlier epidemiological research used this scale among Saudi university students (31–34). The response choice for this scale was a six-point Likert scale (i.e., always, usually, often, sometimes, seldom, and never) and it included 26 items.

Except for 26, all items received scores of 3, 2, and 1 for ‘always’, ‘usually’ and ‘often’, respectively, and 0 for ‘sometimes’, ‘rarely’ and ‘never’. Reverse scoring was applied for the item number 26. The total score range from 0 to 78. Students who scored ≥ 20 indicating that they have disordered eating attitudes (35).

In addition, participants’ food preferences for 10 food items were examined using the procedures used by Siegrist et al. (36) and Sahrin et al. (5). The following question was asked on a six-point Likert scale (“do not like at all” to “like very much”): “How much do you like the following foods?” The food items were: (i) whole grain bread, (ii) vegetables, (ii) fruits, (iv) fish and sea food, (v) milk and dairy products, (vi) red meats, (vii) soft drinks, (viii) processed food, (ix) chocolate and candies, and (x) snacks, chips and nuts.

Food neophobia was the dependent variable of this investigation. The Food Neophobia Scale (FNS, 10 items), first developed by Pliner and Hobden (7), was employed to quantify food neophobia (7). To comply it with the country perspective and population groups, an updated modified version of FNS was used in this investigation. This study adhered to the earlier studies that evaluated food neophobia in university students (5, 18).

The Likert scale used in the FNS was 7 points (1 = strongly disagree and 7 = strongly agree). Individuals’ scores for each item were added up, and the following items had their scores reversed: 1, 4, 6, 9, and 10. The total score range from 10 to 70, where a greater number indicates a higher degree of neophobia towards food. As there is no set cut-off score for dividing individuals into “food neophilics” and “food nephobics” according to their FNS score, many studies have utilized the mean or median FNS score as the cut-off value (37–39). In present study, food neophobia score had a non-normal distribution; therefore, the cut-off point was set at the median value (median FNS score = 37.0). Students those who scored higher than the median were considered to have food neophobia.

### 2.6 Statistical analysis

The analysis of data was performed using SPSS software, version 23.0, with a two-tailed *p*-value of <0.05 being highlighted statistically significant for all tests. Descriptive statistics, chi-square test, correlation and logistic regression analysis were performed where the data supported the assumptions of the respective analysis. The chi-square test was employed to observe the hypothetical association of food neophobia (yes vs. no) by participants’ sociodemographic, health and behavior-related variables. A Spearman’s correlation test was applied to observe the connection between outcome variable and food preferences. Unadjusted and adjusted binary regression analysis were performed to identify the predictors of food neophobia. The adjusted regression model was fitted according to the Hosmer and Lemeshow criterion, and multicollinearity was checked using the variance inflation factor (Table 1). Moreover, a reliability analysis was undertaken to ensure that the scales utilized in this study were internally consistent. The Cronbach’s alpha for the EAT-26 and FNS was 0.73 and 0.81, respectively, confirming satisfactory internal consistency. The results of adjusted regression model fitness test and reliability analysis of food neophobia scale for the present study are presented in Table 1.

**Table 1:** The result of adjusted regression model fitness test and reliability analysis of food neophobia scale for the present study.

Adjusted regression model fitness test		
Criteria	Adjusted regression model	
	Statistics	Remark
(i) Multicollinearity test	Mean VIF: 1.79	Multicollinearity absent.
	Minimum value: 1.102	
	Maximum value: 2.13	
(i) Hosmer and Lemeshow test	Chi-square value: 12.360	Model fitted to run.
	Degree of freedom (df):8	
	*p* value: 0.136	

Reliability analysis of food neophobia			
Items	Mean (SD)	Cronbach’s Alpha (α) if item deleted	Overall reliability statistics
I am constantly sampling new and different foods	3.59 (1.97)	0.794	Cronbach’s α = 0.813
I do not trust new foods	3.53 (1.69)	0.719	
If I do not know what a food is, I will not try it	5.05 (1.91)	0.798	
I like foods from different cultures/districts	3.04 (1.63)	0.899	
Ethnic food looks weird to eat	3.58 (1.77)	0.706	
At dinner parties, I will try new foods	3.04 (1.52)	0.804	
I am afraid to eat things I have never had before	4.02 (1.89)	0.797	
I am very particular about the foods I eat	4.48 (1.98)	0.797	
I will eat almost anything	3.86 (2.09)	0.795	
I like to try ethnic restaurants	3.33 (1.83)	0.797	

## 3 Results

This study included 480 university students with an average age of 22 years (SD: 2.13 and age range: 18 to 25 years). More than half of the respondents were female (52.1%). A higher proportion of the surveyed students were from education discipline (42.7%). One-third of the participants’ (36.0%) monthly family income was between 10,001–15,000 Saudi Arabian Riyal (SAR). Nearly 20% of the participants reported themselves as overweight/obese. Only one-fifth of the participants (19.8%) reported engaging in regular physical exercise. Only 10% of the participants had a smoking habit. A quarter of the participants (26.7%) had a prior experience to get sick after consuming new foods. Above one-third of the study participants had food allergy (35.6%) and had taken dietary supplements (36.5%). Approximately 29% of the respondents had disordered eating attitudes. Table 2 provides comprehensive details on the participants’ sociodemographic, health, and behavioral features.

**Table 2:** Sociodemographic, health and behavioral data of study participants (*N* = 480).

Variables	Categories	Frequency (%)
Gender	Male	230 (47.9)
	Female	250 (52.1)
Age (in years)	18–21	132 (27.5)
	22–25	235 (49.0)
	>25	123 (23.5)
Study discipline	Medicine	76 (15.8)
	Education	205 (42.7)
	Engineering	167 (34.8)
	Science	32 (6.7)
Mother education	Illiterate	68 (14.2)
	Elementary	99 (20.6)
	Intermediate	140 (29.2)
	Secondary	90 (18.8)
	University	83 (17.3)
Father education	Illiterate	40 (8.3)
	Elementary	69 (14.4)
	Intermediate	91 (19.0)
	Secondary	115 (24.0)
	University	165 (34.4)
Current living area	Rental house	257 (53.5)
	Own house	223 (46.5)
Monthly family income (SAR)	<5,000	78 (16.3)
	5,001–10,000	123 (25.6)
	10,001–15,000	173 (36.0)
	>15,000	106 (22.1)
Body mass index	Underweight	80 (16.7)
	Normal weight	298 (62.15)
	Overweight/obese	102 (21.3)
Regular physical exercise	Yes	95 (19.8)
	No	385 (80.2)
Smoking status	Yes	50 (10.4)
	No	430 (89.6)
Food allergy	Yes	171 (35.6)
	No	309 (64.4)
Suffered from anemia	Yes	54 (13.3)
	No	416 (86.7)
Sickness after consuming new foods	Yes	128 (26.7)
	No	352 (73.3)
Taking dietary supplements	Yes	175 (36.5)
	No	305 (63.5)
Disordered eating attitudes	Yes	136 (28.3)
	No	344 (71.7)

The median value of food neophobia score was 37.0 on a range of 10 to 70 (interquartile range: 21). Almost half of the participants exhibited food neophobia based on the FNS (49.6%). The Chi-square test, as depicted in Table 3, shows that participants’ food neophobia was significantly associated with food allergy (*p* < 0.001), experience of illness after consuming new foods (*p* < 0.001), taking dietary supplements (*p* < 0.001) and disordered eating attitudes (*p* = 0.032).

**Table 3:** Bivariate distribution of food neophobia by the explanatory variables and unadjusted regression estimate.

Variables	Food neophobia		*p* value^†^	Unadjusted estimate	
	Yes	No		OR [95%CI]	*p* value
Gender	238 (49.6%)	242 (50.4%)			
Male	109 (47.4%)	121 (52.6%)	0.357	Ref.	
Female	129 (51.6%)	121 (48.4%)		1.18 [0.83–1.69]	0.357
Age (in years)			0.283		
18–21	59 (44.7%)	73 (55.3%)		0.67 [0.40–1.10]	0.113
22–25	117 (49.8%)	118 (50.2%)		0.82 [0.52–1.28]	0.375
>25	62 (54.9%)	51 (45.1%)		Ref.	
Study discipline			0.337		
Medicine	41 (53.9%)	35 (46.1%)		0.80 [0.35–1.85]	0.605
Education	93 (45.4%)	112 (54.6%)		0.57 [0.27–1.21]	0.143
Engineering	85 (50.9%)	82 (49.1%)		0.71 [0.33–1.53]	0.381
Science	19 (59.4%)	13 (40.6%)		Ref.	
Mother education			0.580		
Illiterate	32 (47.1%)	36 (52.9%)		Ref.	
Elementary	48 (48.5%)	51 (52.5%)		1.06 [0.57–1.97]	0.856
Intermediate	70 (50.0%)	70 (50.0%)		1.13 [0.63–2.01]	0.691
Secondary	51 (56.7%)	39 (43.3%)		1.47 [0.78–2.77]	0.232
University	37 (44.6%)	46 (55.4%)		0.91 [0.48–1.72]	0.761
Father education			0.237		
Illiterate	20 (50.0%)	20 (50.0%)		Ref.	
Elementary	26 (37.7%)	43 (62.3%)		0.61 [0.28–1.33]	0.211
Intermediate	48 (52.7%)	43 (47.3%)		1.12 [0.53–2.35]	0.772
Secondary	63 (54.8%)	52 (45.2%)		1.21 [0.59–2.49]	0.602
University	81 (49.1%)	84 (50.9%)		0.96 [0.48–1.92]	0.918
Current living			0.907		
Rental house	129 (50.2%)	128 (49.8%)		1.02 [0.68–1.53]	0.925
Own house	109 (48.9%)	114 (51.1%)		Ref.	
Monthly family income (in SAR)			0.466		
<5,000	42 (53.8%)	36 (46.2%)		1.03 [0.56–1.80]	0.992
5,001–10,000	55 (44.7%)	68 (55.3%)		0.70 [0.41–1.17]	0.172
10,001–15,000	84 (48.6%)	89 (51.4%)		0.81 [0.50–1.32]	0.398
>15,000	57 (53.8%)	49 (46.2%)		Ref.	
Body mass index			0.687		
Underweight	43 (53.8%)	37 (46.3%)		1.24 [0.76–2.04]	0.389
Normal weight	144 (48.3%)	154 (51.7%)		Ref.	
Overweight/obesity	51 (50.0%)	51 (50.0%)		1.07 [0.68–1.68]	0.770
Regular physical exercise			0.630		
Yes	45 (47.4%)	50 (52.6%)		0.90 [0.57–1.40]	0.630
No	193 (50.1%)	192 (49.9%)		Ref.	
Smoking			0.509		
Yes	27 (54.0%)	23 (46.0%)		1.22 [0.68–2.19]	0.510
No	211 (49.1%)	219 (50.9%)		Ref.	
Food allergy			**0.000**		
Yes	124 (72.5%)	47 (27.5%)		4.51 [3.00–6.78]	**0.000**
No	114 (36.9%)	195 (63.1%)		Ref.	
Suffered from anemia			0.734		
Yes	33 (51.6%)	31 (48.4%)		1.09 [0.65–1.86]	0.734
No	205 (49.3%)	211 (50.7%)		Ref.	
Sickness after consuming new foods			**0.000**		
Yes	90 (70.3%)	38 (29.7%)		3.27 [2.11–5.04]	**0.000**
No	148 (42.0%)	204 (58.0%)		Ref.	
Taking dietary supplements			**0.000**		
Yes	129 (73.7%)	46 (26.3%)		5.04 [3.35–7.59]	**0.000**
No	109 (35.7%)	196 (64.3%)		Ref.	
Disordered eating attitudes			**0.032**		
Yes	78 (57.4%)	58 (42.6%)		1.55 [1.04–2.31]	**0.033**
No	160 (46.5%)	184 (53.5%)		Ref.	

SAR, Saudi Riyal; OR, Odds ratio; CI, confidence interval, p, probability value. Bolded values indicate statistically significant.

† Indicates *p* value was determined by chi-square test.

Unadjusted binary logistic regression analysis revealed the four predictors of food neophobia. These are: (i) food allergy (crude odds ratio, COR: 4.51, 95% confidence interval (CI): 3.00–6.78, *p* < 0.001), (ii) experience of illness after consuming new foods (COR: 3.27, 95%CI: 2.11–5.04, *p* < 0.001), (iii) taking dietary supplements (COR: 5.04 95%CI: 3.35–7.59, *p* < 0.001) and (iv) disordered eating attitudes (COR: 1.55, 95%CI: 1.04–2.31, *p* = 0.033) (Table 3).

Table 4 demonstrates the adjusted estimated effect of the predictors on having food neophobia using an adjusted binary logistic regression model. Participants who engaged in regular physical exercise had a lower risk of developing food neophobia than their counterparts (adjusted odds ratio, AOR: 0.43, 95%CI: 0.20–0.92, *p* = 0.030). Participants with food allergies showed a greater risk of developing food neophobia compared to those without food allergies (AOR: 4.36, 95%CI: 2.73–6.94, *p* < 0.001). Participants who took dietary supplements had a higher probability of having food neophobia than their fellow counterparts (AOR: 6.76, 95%CI: 3.54–12.90, *p* < 0.001). The likelihood of developing food neophobia was 2.5 times greater among participants exhibiting disordered eating attitudes (AOR: 2.52, 95%CI: 1.27–5.02, *p* = 0.008).

**Table 4:** Factors associated with food neophobia among a sample university students in Saudi Arabia (*N* = 480).

Variables	Adjusted Regression Estimate				
	SE	Wald	OR	95%CI	*p* value
Gender [ref. male]					
Female	0.24	0.01	1.03	0.64–1.66	0.917
Age [ref. >25 years]					
18–21	0.34	1.37	0.67	0.35–1.31	0.242
22–25	0.30	0.39	0.83	0.46–1.50	0.532
Study discipline [ref. science]					
Medicine	0.68	2.10	0.37	0.09–1.42	0.148
Education	0.54	1.29	0.55	0.19–1.56	0.256
Engineering	0.51	0.84	0.63	0.23–1.70	0.358
Mother education [ref. illiterate]					
Elementary	0.58	0.99	1.78	0.57–5.54	0.319
Intermediate	0.52	0.16	1.23	0.44–3.41	0.691
Secondary	0.58	1.63	2.09	0.67–6.50	0.202
University	0.67	0.01	1.05	0.28–3.90	0.945
Father education [ref. illiterate]					
Elementary	0.66	1.41	0.46	0.13–1.66	0.235
Intermediate	0.74	0.09	1.25	0.29–5.29	0.759
Secondary	0.73	0.54	1.70	0.41–7.07	0.463
University	0.65	0.03	0.89	0.26–3.18	0.857
Current living area [ref. own house]					
Rental house	0.32	0.17	0.88	0.47–1,64	0.683
Dormitory	0.33	0.05	1.08	0.56–2.07	0.818
Monthly family income [ref. >15000SAR]					
<5,000 SAR	0.37	0.01	1.04	0.51–2.12	0.921
5,001–10,000 SAR	0.34	1.57	0.66	0.34–1.27	0.211
10,001–15,000	0.31	0.82	0.75	0.41–1.39	0.366
Body mass index [ref. normal]					
Underweight	0.35	0.50	1.28	0.64–2.55	0.480
Overweight/obesity	0.34	0.01	0.98	0.51–1.88	0.946
Regular physical exercise [ref. no]					
Yes	0.39	4.71	0.43	0.20–0.92	0.030*
Smoking [ref. no]					
Yes	0.36	0.44	1.27	0.63–2.54	0.510
Food allergy [ref. no]					
Yes	0.24	38.21	4.36	2.73–6.94	0.000*
Suffered from anemia [ref. no]					
Yes	0.34	0.00	1.01	0.52–1.97	0.983
Sickness after consuming new foods [ref. no]					
Yes	0.34	0.02	0.95	0.49–1.85	0.876
Taking dietary supplements [ref. no]					
Yes	0.33	33.49	6.76	3.54–12.90	0.000*
Disordered eating attitudes [ref. no]					
Yes	0.35	6.96	2.52	1.27–5.02	0.008*

SE, standard error; OR, Odds ratio; CI, confidence interval; p, probability value; SAR, Saudi Arabian riyal (The currency of Saudi Arbia). Asterisk and italic values indicate statistically significant.

The relationship between food neophobia scores and participants’ preferences for different foods is displayed in Table 5. The preferences for fish and sea food (Spearman’s correlation coefficient, r_s_ = −0.150, *p* = 0.001), milk and dairy products (r_s_ = −0.309, *p* < 0.001), chocolate and candies (r_s_ = −0.329, *p* < 0.001) and snacks, chips, and nuts (r_s_ = −0.166, *p* < 0.001) were significantly negatively correlated with food neophobia. The negative coefficient values for all the food categories mean that as food neophobia increases, the preference for these foods decreases. Specifically, the weak but statistically significant negative correlation between food neophobia and preference for fish and seafood suggests that fish and seafood are somewhat more accepted even among students with higher food neophobia, possibly because they are common in certain cultural or dietary contexts. However, the correlation still indicates that, overall, higher neophobia is associated with lower preference for these foods, consistent with the broader trend observed across the other food categories.

**Table 5:** Relationship between food neophobia scores and preferences for different food items among study participants (*N* = 480).

Food items	Spearman’s correlation coefficient	*p* value (2-tailed)
Whole grain bread	0.054	0.241
Vegetables	−0.078	0.087
Fruits	−0.049	0.289
Fish and sea food	−0.150	**0.001**
Milk and dairy products	−0.309	**0.000**
Red Meat	0.023	0.616
Soft drinks	0.097	0.063
Processed foods	−0.010	0.819
Chocolates and candies	−0.329	**0.000**
Snacks, chips and nuts	−0.166	**0.000**

Bolded values indicate significant correlation.

## 4 Discussion

The present study reported approximately half of the university students had food neophobia (49.6%). The findings reveal that a considerable proportion of university students in Saudi Arabia exhibit food neophobia. A high level of food neophobia among university students has also been seen in China (18) and Bangladesh (5). One possible rationale is that university age is an important stage in an individual’s life when they gain independence and a concentrated lifestyle, which can foster the emergence of unhealthy eating habits, eating disorders, low physical activity and mental health disturbances. The findings suggest additional nationwide research to reveal a broader picture of food neophobia among university students as well as other population groups such as adolescents and young adults in Saudi Arabia.

This study revealed that students who engaged in regular physical activity had a lower risk of developing food neophobia. Physical activity and diet are important indicators of public health status that interact with one another. Physical activity has been found to improve healthy dietary choices and assist regulate eating behaviors (40). Regular physical activity (moderate-to-vigorous) has been linked to a preference for low-fat/low-energy foods and a decreased desire for high-fat foods (41). Physically active individuals place a higher priority on the nutritional and health benefits of foods in their food choices (42). Furthermore, there is convincing evidence that regular physical activity can help avoid certain chronic diseases (e.g., diabetes, obesity, etc.) and early death (43). Generally, engaging in regular physical exercise can enhance psychological resilience and receptivity to new experiences. This change in mindset may extend to dietary habits, making people more willing to consume new and varied foods.

This also found that students with food allergies had a higher chance of having food neophobia than those without food allergies. This finding is consistent with a prior study (5). Individuals with food allergies may feel anxious about the possibility of adverse reactions. This anxiety or fear can cause individuals to avoid trying new meals entirely, increasing food neophobia. Food neophobia is undoubtedly triggered by the restriction of dietary options owing to food allergies (44).

Dietary supplement consumption and food neophobia are interrelated concepts in nutrition and psychology. The current study showed university students who took dietary supplements had a greater chance of having food neophobia compared to their counterparts. This finding is supported by rising consumption patterns of dietary supplement in Saudi Arabia (45). In general, peoples consume dietary supplements to enhance their overall health (45). However, individuals who largely depend on food supplements to achieve their nutritional demands may be less inclined to consume organic and diversified diets. Individuals with high food neophobia, on the other hand, often exhibited strong preferences for familiar foods and may fear trying new or different items, including whole foods that contain essential nutrients. This reluctance may drive people to relay more on dietary supplements to meet or compensate their nutritional requirements, as they seek familiar and convenient alternatives. Hence, further follow-up studies are recommended to explore the directional relationship between dietary supplement consumption and food neophobia.

This study demonstrated that students with disordered eating attitudes had a higher likelihood of developing food neophobia. This finding is comparable with a recent study, representing a positive strong correlation between food neophobia and avoidant/restrictive food intake disorder (46). The comparison of this study’s finding with Białek-Dratwa et al.’s study (46) is justified by the fact that the current assessed disordered eating attitudes by EAT-26, which truly represent restrictive eating pathology or disorder (47). People with restrictive eating habits or disordered eating attitudes may have a negative body image and fear losing control or gaining weight. This can lead to avoidance behavior, where people restrict their diet to familiar, often less diverse options. More longitudinal research is needed to deeper understand this association, which could help inform interventions aiming at resolving both disordered eating and food neophobia to promote healthy attitudes towards food and eating behaviors.

Food neophobia affects a person’s preference for foods across different food groups (see Table 4). Similar relationship was found in a recent study conducted in Bangladeshi university students (5). Sahrin et al. (5) showed that food neophobia was negatively correlated with the liking of vegetables, chocolate and candies and chips, nuts, and snacks. The present study’s findings suggest that neophobic university students exhibit some dislike towards the consumption of fish and sea food, and milk and milk products. According to a nationally representative survey, only 44.7% of people in Saudi Arabia followed dietary guidelines for fish, which is worrying for acquiring high-quality proteins, micronutrients, and minerals (48). Evidence suggests that young adults in Saudi Arabia consume a high amount of processed meals and sugar-sweetened beverages (48). Since female participants are predominant in this study—who tend to be more food neophobic (prevalence of food neophobia among female vs. male = 51.6% vs. 47.4%) and often avoid sweets and fatty foods (49, 50)—it’s not unusual that there was a negative correlation between food neophobia and liking for chocolate, candies, chips, nuts, and snacks. Similar justification is provided by Sahrin et al. (5) for justifying above-mentioned relationship. However, it has not been explicitly clarified whether the avoidance of these foods is due to neophobia or other reasons such as physical appearance concerns. To fully understand the motivations behind avoiding these foods, additional research is needed to separate the effects of food neophobia from other factors, such as dietary preferences or body image concerns.

### 4.1 Policy implications of the findings

This study was one of the very first studies that explore university students’ food neophobia in Saudi Arabia; hence the findings can be used as baseline statistics for future research initiatives. Because a significant degree of food neophobia was found among university students in the country, nutrition counselling programs should focus on food neophobia as a potential roadblock to a healthy diet. University administrators should offer comprehensive nutrition education programs that focus on physical exercise, food allergies, dietary supplement intake, and eating disorders so that students can overcome food neophobia and eat a balanced and varied diet. Policymakers can use our findings to further improve health and nutrition policies in Saudi Arabia.

### 4.2 Limitations of this study

The cross-sectional study design hinders the establishment of a causal relationship. Because this study was limited to two universities, the results cannot be generalized to other areas or age groups in Saudi Arabia. Moreover, self-reporting biases can potentially present in the samples. This study did not undertake any sub-group analysis. A sub-group analysis considering factors such as prior diseases, personal medical, social and medical background, or non-student population groups (as part of a case–control analysis) could have provided the evidence-based conclusions and offered more targeted insights for different demographic or clinical subgroups.

## 5 Conclusion

A higher prevalence of food neophobia was observed among sampled university students in Saudi Arabia. Several factors such as regular physical exercise, food allergies, dietary supplement consumption and disordered eating attitudes were found to be associated with food neophobia. Future longitudinal studies are suggested to deeper understanding the factors that influence food neophobia in Saudi university students. Furthermore, to provide empirical evidence on food neophobia, additional research with large and representative samples could be performed in other regions of Saudi Arabia.

## Data Availability

The raw data supporting the conclusions of this article will be made available by the author, without undue reservation.
