# Supplementary material for: Food neophobia among university students in Saudi Arabia: a cross-sectional survey of prevalence and predictor analysis
Source: Front Public Health. 2025 Apr 1;13:1571899. doi: 10.3389/fpubh.2025.1571899 (PMC11996773; doi:10.3389/fpubh.2025.1571899)
Supplement: Supplementary file 1 [file Table_1.DOCX]

Supplementary file 1: **Survey questionnaire on “Food neophobia among university students in Saudi Arabia”**

**# Section 1:** Sociodemographic and health-related information

| Variables | Categories |
| --- | --- |
| Gender | 1) Male |
|  | 2) Female |
| Age (in years) | 1)18-21 |
|  | 2) 22-25 |
|  | 3) >25 |
| Study discipline | 1) Medicine |
|  | 2) Education |
|  | 3) Engineering |
|  | 4) Science |
| Mother education | 1) Illiterate |
|  | 2) Elementary |
|  | 3) Intermediate |
|  | 4) Secondary |
|  | 5) University |
| Father education | 1) Illiterate |
|  | 2) Elementary |
|  | 3) Intermediate |
|  | 4) Secondary |
|  | 5) University |
| Current living area | 1) Rental house |
|  | 2) Own house |
| Monthly family income (SAR) | 1) <5000 |
|  | 2) 5001-10000 |
|  | 3) 10001-15000 |
|  | 4) >15000 |
| Body mass index | 1) Underweight |
|  | 2) Normal weight |
|  | 3) Overweight/obese |
| Regular physical exercise | 1) Yes |
|  | 2) No |
| Smoking status | 1) Yes |
|  | 2) No |
| Food allergy | 1) Yes |
|  | 2) No |
| Suffered from anemia | 1) Yes |
|  | 2) No |
| Sickness after consuming new foods | 1) Yes |
|  | 2) No |
| Taking dietary supplements | 1) Yes |
|  | 2) No |

**# Section 2:** The Eating Attitude Test-26 (EAT-26) questionnaire

|  |  | **Always** | **Usually** | **Often** | **Sometimes** | **Rarely** | **Never** |
| --- | --- | --- | --- | --- | --- | --- | --- |
| **1.** | Am terrified about being overweight. |  |  |  |  |  |  |
| **2.** | Avoid eating when I am hungry. |  |  |  |  |  |  |
| **3.** | Find myself preoccupied with food |  |  |  |  |  |  |
| **4.** | Have gone on eating binges where I feel that I may not be able to stop. |  |  |  |  |  |  |
| **5.** | Cut my food into small pieces. |  |  |  |  |  |  |
| **6.** | Aware of the calorie content of foods that I eat. |  |  |  |  |  |  |
| **7.** | Particularly avoid food with a high carbohydrate content (i.e. bread, rice, potatoes, etc.) |  |  |  |  |  |  |
| **8.** | Feel that others would prefer if I ate more. |  |  |  |  |  |  |
| **9.** | Vomit after I have eaten. |  |  |  |  |  |  |
| **10.** | Feel extremely guilty after eating. |  |  |  |  |  |  |
| **11.** | Am preoccupied with a desire to be thinner. |  |  |  |  |  |  |
| **12.** | Think about burning up calories when I exercise. |  |  |  |  |  |  |
| **13.** | Other people think that I am too thin. |  |  |  |  |  |  |
| **14.** | Am preoccupied with the thought of having fat on my body. |  |  |  |  |  |  |
| **15.** | Take longer than others to eat my meals. |  |  |  |  |  |  |
| **16.** | Avoid foods with sugar in them. |  |  |  |  |  |  |
| **17.** | Eat diet foods. |  |  |  |  |  |  |
| **18.** | Feel that food controls my life. |  |  |  |  |  |  |
| **19.** | Display self-control around food. |  |  |  |  |  |  |
| **20** | Feel that others pressure me to eat. |  |  |  |  |  |  |
| **21.** | Give too much time and thought to food. |  |  |  |  |  |  |
| **22.** | Feel uncomfortable after eating sweets. |  |  |  |  |  |  |
| **23.** | Engage in dieting behavior. |  |  |  |  |  |  |
| **24.** | Like my stomach to be empty. |  |  |  |  |  |  |
| **25.** | Have the impulse to vomit after meals. |  |  |  |  |  |  |
| **26.** | Enjoy trying new rich foods. |  |  |  |  |  |  |

**# Section 3:** Participants’ food preferences

How much do you like the following foods?

| Food items | Do not like at all | Dislike slightly | Neither like or dislike | Like slightly | Like very much |
| --- | --- | --- | --- | --- | --- |
| 1. Whole grain bread |  |  |  |  |  |
| 2. Vegetables |  |  |  |  |  |
| 3. Fruits |  |  |  |  |  |
| 4. Fish and sea food, |  |  |  |  |  |
| 5.Milk and dairy products, |  |  |  |  |  |
| 6. Red meats |  |  |  |  |  |
| 7. Soft drinks, |  |  |  |  |  |
| 8. Processed food, |  |  |  |  |  |
| 9. Chocolate and candies, |  |  |  |  |  |
| 10. snacks, chips and nuts. |  |  |  |  |  |

**# Section 4:** Assessment of food neophobia

| 1. **I am constantly sampling new and different foods (R)** | 1. Strongly disagree 2. Disagree 3. Somewhat disagree 4. Neither agree nor disagree | 1. Somewhat agree 2. Agree 3. Strongly agree |  |
| --- | --- | --- | --- |
| 1. **I don’t trust new foods** | 1. Strongly disagree 2. Disagree 3. Somewhat disagree 4. Neither agree nor disagree | 1. Somewhat agree 2. Agree 3. Strongly agree |  |
| 1. **If I don’t know what a food is, I won’t try it** | 1. Strongly disagree 2. Disagree 3. Somewhat disagree 4. Neither agree nor disagree | 1. Somewhat agree 2. Agree 3. Strongly agree |  |
| 1. **I like foods from different cultures/districts (R)** | 1. Strongly disagree 2. Disagree 3. Somewhat disagree 4. Neither agree nor disagree | 1. Somewhat agree 2. Agree 3. Strongly agree |  |
| 1. **Ethnic food looks weird to eat** | 1. Strongly disagree 2. Disagree 3. Somewhat disagree 4. Neither agree nor disagree | 1. Somewhat agree 2. Agree 3. Strongly agree |  |
| 1. **At dinner parties, I will try new foods (R)** | 1. Strongly disagree 2. Disagree 3. Somewhat disagree 4. Neither agree nor disagree | 1. Somewhat agree 2. Agree 3. Strongly agree |  |
| 1. **I am afraid to eat things I have never had before** | 1. Strongly disagree 2. Disagree 3. Somewhat disagree 4. Neither agree nor disagree | 1. Somewhat agree 2. Agree 3. Strongly agree |  |
| 1. **I am very particular about the foods I eat** | 1. Strongly disagree 2. Disagree 3. Somewhat disagree 4. Neither agree nor disagree | 1. Somewhat agree 2. Agree 3. Strongly agree |  |
| 1. **I will eat almost anything (R** | 1. Strongly disagree 2. Disagree 3. Somewhat disagree 4. Neither agree nor disagree | 1. Somewhat agree 2. Agree 3. Strongly agree |  |
| 1. **I like to try ethnic restaurants (R)** | 1. Strongly disagree 2. Disagree 3. Somewhat disagree 4. Neither agree nor disagree | 1. Somewhat agree 2. Agree 3. Strongly agree |  |
